# Phylogeography of *Bulinus truncatus* (Audouin, 1827) (Gastropoda: Planorbidae) in Selected African Countries

**DOI:** 10.3390/tropicalmed3040127

**Published:** 2018-12-19

**Authors:** Eniola M. Abe, Yun-Hai Guo, Haimo Shen, Masceline J. Mutsaka-Makuvaza, Mohamed R. Habib, Jing-Bo Xue, Nicholas Midzi, Jing Xu, Shi-Zhu Li, Xiao-Nong Zhou

**Affiliations:** 1 National Institute of Parasitic Diseases (NIPD), Chinese Centre for Disease Control and Prevention, Shanghai 200025, China; guoyunhaigy@163.com (Y.G.); shenhm@nipd.chinacdc.cn (H.S.); xuejb@nipd.chinacdc.cn (J.-B.X.); xujing@nipd.chinacdc.cn (J.X.); lisz@chinacdc.cn (S.-Z.L.); 2 Department of Medical Microbiology, College of Health Sciences, University of Zimbabwe, Harare 00263, Zimbabwe; mascelinejeni@gmail.com (M.M.-M.); midzinicholas@gmail.com (N.M.); 3 Medical Malacology Laboratory, Theodor Bilharz Research Institute, Giza 12411, Egypt; m_ramadanhabib@yahoo.com

**Keywords:** phylogeography, *Bulinus truncatus*, planorbidae, Africa

## Abstract

The transmission of some schistosome parasites is dependent on the planorbid snail hosts. *Bulinus truncatus* is important in urinary schistosomiasis epidemiology in Africa. Hence, there is a need to define the snails’ phylogeography. This study assessed the population genetic structure of *B. truncatus* from Giza and Sharkia (Egypt), Barakat (Sudan) and Madziwa, Shamva District (Zimbabwe) using mitochondrial cytochrome oxidase subunit 1 gene (COI) and internal transcribed spacer 1 (ITS 1) markers. COI was sequenced from 94 *B. truncatus* samples including 38 (Egypt), 36 (Sudan) and 20 (Zimbabwe). However, only 51 ITS 1 sequences were identified from Egypt (28) and Sudan (23) (because of failure in either amplification or sequencing). The unique COI haplotypes of *B. truncatus* sequences observed were 6, 11, and 6 for Egypt, Sudan, and Zimbabwe, respectively. Also, 3 and 2 unique ITS 1 haplotypes were observed in sequences from Egypt and Sudan respectively. Mitochondrial DNA sequences from Sudan and Zimbabwe indicated high haplotype diversity with 0.768 and 0.784, respectively, while relatively low haplotype diversity was also observed for sequences from Egypt (0.334). The location of populations from Egypt and Sudan on the *B. truncatus* clade agrees with the location of both countries geographically. The clustering of the Zimbabwe sequences on different locations on the clade can be attributed to individuals with different genotypes within the population. No significant variation was observed within *B. truncatus* populations from Egypt and Sudan as indicated by the ITS 1 tree. This study investigated the genetic diversity of *B. truncatus* from Giza and Sharkia (Egypt), Barakat area (Sudan), and Madziwa (Zimbabwe), which is necessary for snail host surveillance in the study areas and also provided genomic data of this important snail species from the sampled countries.

## 1. Background

The snail intermediate hosts of the genus *Bulinus* play active roles in the epidemiology of urinary schistosomiasis. The schistosome parasites depend on these snails for the development of the asexual phase of their life cycle before the cercariae are released into the water bodies to look for unsuspecting human hosts for penetration, where they continue the sexual phase of their development [1,2,3,4].

Members of the genus *Bulinus* are hermaphroditic planorbid snails, and this genus includes 37 recognized species distributed in the tropic and sub-tropic regions of the world including Africa, Mediterranean countries, and parts of the Middle East [5]. They differ in their interaction with schistosome parasites and some are involved in the transmission of human and animal schistosomiasis [6].

These important snail species inhabit various types of freshwater bodies such as streams, ponds, rivers, and irrigation canals [7]. The genetic structure of snail hosts is mostly determined by their habitat distribution, which is largely influenced by the spatial and temporal fluctuations in water availability [8,9] leading to population bottlenecks [5].

Snails belonging to the *Bulinus* group have a great capacity to rapidly increase their population size through cross- or self-fertilization, but *B. truncatus* has a preference for self-fertilization [10]. Selfing and population bottlenecks increase genetic differentiation among snail population but reduce the amount of genetic diversity within a population [11].

Whilst morphological identification of snails helps with identifying snails at group or genus level, it cannot give further insights about their interaction with the parasites [12].

Assessment of snail hosts population structure using molecular markers and other genetic tools creates a robust system for species identification and differentiation [8,12,13,14,15,16,17]. This provides useful information about their genetic diversity and detailed elucidation of the host–parasite relationship [4], which can be applied to target effective integrated schistosomiasis control strategies in most endemic areas [18].

The use of different markers such as COI, microsatellites and ITS 1, has helped to achieve identification of *B. truncatus* sampled from few African countries including Senegal, Niger, Tanzania, Burkina Faso, and Cameroon [11,12]. Studies have also observed strong population subdivision and low diversity for hermaphroditic freshwater snails including *B. truncatus* [11,19,20,21,22,23].

It is, therefore, imperative to provide information on the diversity of important snail hosts including *B. truncatus* through assessing their phylogenic status in most countries endemic for schistosomiasis across Africa, to further improve our understanding about their phylogenetic relationships as well as the disease epidemiology.

This study provided information on the phylogeography of *B. truncatus* populations from Giza and Sharkia (Egypt), Barakat area (Sudan), and Madziwa, Shamva District (Zimbabwe), using partial mitochondrial DNA cytochrome oxidase subunit I (COI) and internal transcribed spacer 1 (ITS 1) to determine their phylogenetic relationship, which is important for epidemiological investigation and snail hosts surveillance.

## 2. Materials and Methods

### 2.1. Sample Collection

*Bulinus* snails were collected from different locations in freshwater bodies at Giza and Sharkia governorates (Egypt), Barakat area (Sudan) and Madziwa area, Shamva District (Zimbabwe). Snail sampling was done at selected sites along water bodies; these included water contact sites where people swim, carry out fishing activities, collect water for domestic purposes, bathing, and washing clothes and utensils. Sites with no apparent human water contact activities were also visited for snail collection. A total of 134 *Bulinus* snails was assessed from different locations across the three countries.

**Figure 1:**
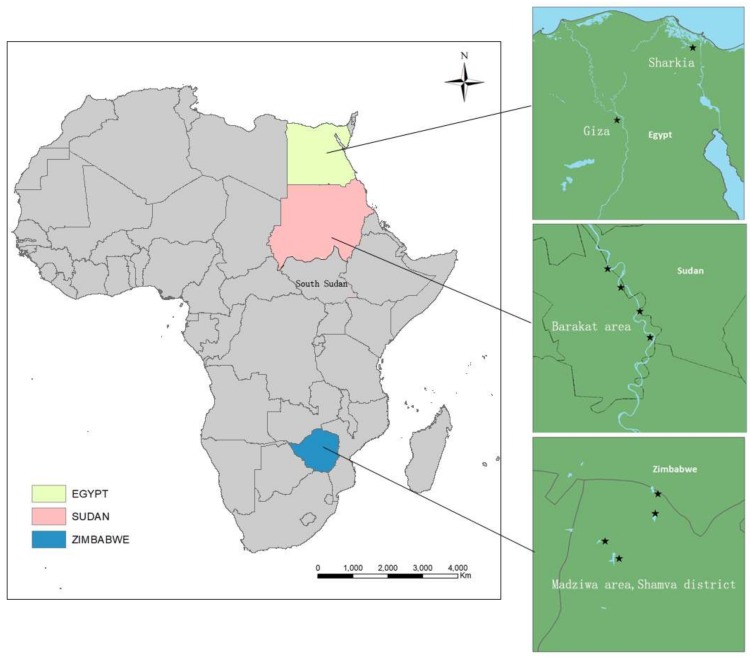
Map of Africa showing countries where snail samples were collected.

The snails were identified phenotypically using shell morphology [24]. Snails were then preserved in absolute ethanol. Information that includes snail collection and geographic coordinates of the study areas are shown in Table 1. A map of study areas is shown in Figure 1.

### 2.2. Sample Preparation and DNA Extraction

The specimens were recounted, identified by morphological characters, and re-spirited (absolute ethanol) upon arrival at the National Institute of Parasitic Diseases, Shanghai, schistosomiasis laboratory [25]. Specimens were placed in TE buffer (10 mM Tris, 0.1 mM EDTA) pH 7.4 for 1 h to remove the remaining alcohol from within the tissue, which might interfere with subsequent extraction techniques. Total genomic DNA was isolated from head-foot snail tissue using the DNeasy Blood and Tissue kit (Qiagen, Crawley, UK) according to the manufacturer’s instructions. DNA was eluted into 200 μL AE buffer. The snails’ genomic DNA concentration was quantified using the Nanodrop ND-1000 Spectrophotometer (Nanodrop Technologies Inc., Thermo Fisher Scientific, Wilmington, DE, USA).

### 2.3. Polymerase Chain Reaction (PCR) Amplification of COI and ITS 1 Fragments

IllustraPuRe Taq Ready-To-Go PCR beads (GE Healthcare) were used for the amplification of the COI and ITS 1 fragments using the methods outlined in Kane et al. [12] with 0.4 µM each of Bulcox 5 (5′CCT TTA AGA GGN CCT ATT GC 3′) (forward primer) and Bulcox 14 (5′GGA AAT CAG TAM AYA AAA CCA GC 3′) (reverse primer) while ETTS10 (5′ GCA TAC TGC TTT GAA CAT CG 3′) (forward primer) and ETTS1(5′GC TTA AGT TCA GCG GGT 3′) (reverse primer) were used for *B. truncatus* amplification. A DNA template of 1 µL was added to each tube that contained 22 µL double distilled water, 1 µL each of forward and reverse primers. The total reaction volume was 25 µL. Double distilled water was used as the negative control. PCR amplification of snail genomic DNA was done using Applied Biosystems GeneAmp Thermal Cycler 2700 version 2.08. Cycling conditions for COI and ITS 1 reactions are as follows: one cycle of 95 °C for 5 min, 45 cycles of 95 °C for 30 s, 54.3 °C for 30 s, 72 °C for 45 s and 72 °C for 10 min and one cycle of 95 °C for 5 min, 45 cycles of 95 °C for 30 s, 42 °C for 30 s, 72 °C for 45 s and 72 °C for 10 min respectively. PCR fragments were separated on 1% agarose gel and visualization was performed using a gel documentation and analysis system (UVP, EpiChem II darkroom). Sequencing was performed on an Applied Biosystems 3730XL analyser (Life Technologies, Northumberland, UK).

### 2.4. Phylogenetic Analysis of Sequence Data

Nucleotide sequences were visually edited using Bioedit software v 7.0. [26]. BLAST searches via the National Centre for Biotechnology Information (http://www.ncbi.nlm.nih.gov/) were performed for the obtained sequences against Genbank database to ensure that contaminant sequences had not been obtained by error [27] and aligned with the reference materials [12] using the Clustal W algorithm [28]. We performed the maximum-likelihood analyses for the COI and ITS 1 sequences using the program RAxML [29]. The maximum-likelihood estimates were bootstrapped for 1000 replicates based on the GTRGAMMA substitution model. Downloaded *B. truncatus* sequences deposited in Genbank from Niger (AM286316.2), Senegal (AM921807.1 and AM921806.1) Portugal (AM286314), Italy (AM286312.3), Burkina Faso (AM286315.2), and Tanzania (AM286313.2) [12] were used as reference isolates for COI sequences, while *B. truncatus* isolates from Tanzania (AM921983), Niger (AM921965) [16], and Cameroon (KJ157504.1, KJ157503.1, KJ157500.1, KJ157501.1, KJ157502.1) [13] were used as reference isolates for ITS 1 sequences. Sequence data from other *Bulinus* species on Genbank (detailed information on accession number and origin provided as Supplementary Data) were also included in constructing the maximum likelihood phylogenetic trees. Additionally, *Bulinus forskalii* (AM286306.2) was used as an outgroup for *Bulinus truncatus* group assessed with COI marker. *Bulinus forskalii* (AM921961.1) was used as the outgroup for the *Bulinus truncatus* group assessed with ITS 1 marker.

We also estimated the phylogenetic relationships of the COI and ITS 1 *B. truncatus* dataset using Bayesian inference in MrBayes version 3.2.0 programs [30] (Figures S1 and S2). Prior to Bayesian inference, the best fit nucleotide substitution models (HKY for COI and TrN for ITS 1) were determined using a hierarchical likelihood ratio test in jMODELTEST version 0.1.1 [31]. The posterior probabilities were calculated via 1,000,000 generations using Markov chain Monte Carlo (MCMC) simulations, and the chains were sampled every 1000 generations. At the end of this run, the average standard deviation of split frequencies was below 0.01, and the potential scale reduction factor was reasonably close to 1.0 for all parameters. A consensus tree was summarized and visualized in FigTree version 1.4.3 [32].

*B. truncatus* sequences from the three populations, reference isolates, and other *Bulinus* species sequences used for constructing ML trees were repeated for the construction of the Bayes ML trees Clade comprising *B. forskalii* (AM286308, AM286293.2, and AM286306.2) was used as the outgroup for COI *B. truncatus* sequences while *B. forskalii* (AM921961.1) was used the outgroup for ITS 1 *B. truncatus* sequences.

The minimum spanning tree was built using NETWORK 5.0.0.0 [33] (Figure S3). We built the network to support the COI ML tree and it showed the torso of the genetic structure.

DNA sequences have been submitted to the National Centre for Biotechnology Information Archive with accession numbers MG759386–MG759479 (*B. truncatus* group assessed with COI marker) and MG757840-757890 (*B. truncatus* group assessed with ITS 1 marker).

### 2.5. Determination of Haplotype and Nucleotide Diversity

The level of sequence diversity, which includes number of haplotype (h), haplotype diversity (hd), nucleotide diversity (π), Tajima’s D (D), and theta per site statistics, were calculated for *B. truncatus* populations assessed with both COI and ITS 1 markers in Arlequin software version 3.5 [34]. In addition, we compared Fst of *B. truncatus* studied populations using Arlequin software version 3.5 [34].

## 3. Results 

### 3.1. Phylogeny

Altogether, 94 individual snail samples including 38 (Giza and Sharkia; Egypt), 36 (Barakat area; Sudan), and 20 (Madziwa, Shamva District; Zimbabwe) were successfully sequenced at the COI region (Table 2), and 51 including 28 (Egypt) and 23 (Sudan) were sequenced at the ITS 1 locus (Table 2), because of failure in either amplification or sequencing. Following the sequencing alignment and trimming of all the sequences, the final fragments of 737 bp (COI) and 580 bp (ITS 1) were obtained. Among these, 6, 11, and 6 unique COI haplotypes of *B. truncatus* sequences were observed from Giza and Sharkia (Egypt), Barakat area (Sudan) and Madziwa, Shamva district (Zimbabwe), respectively (Table 2). In Egypt and Sudan, respectively, 3 and 2 unique ITS 1 haplotypes of *B. truncatus* sequences were observed (Table 2). No information on *B. truncatus* ITS 1 sequences from Zimbabwe was recorded in this study due to failure in either amplification or sequencing.

**Figure 2:**
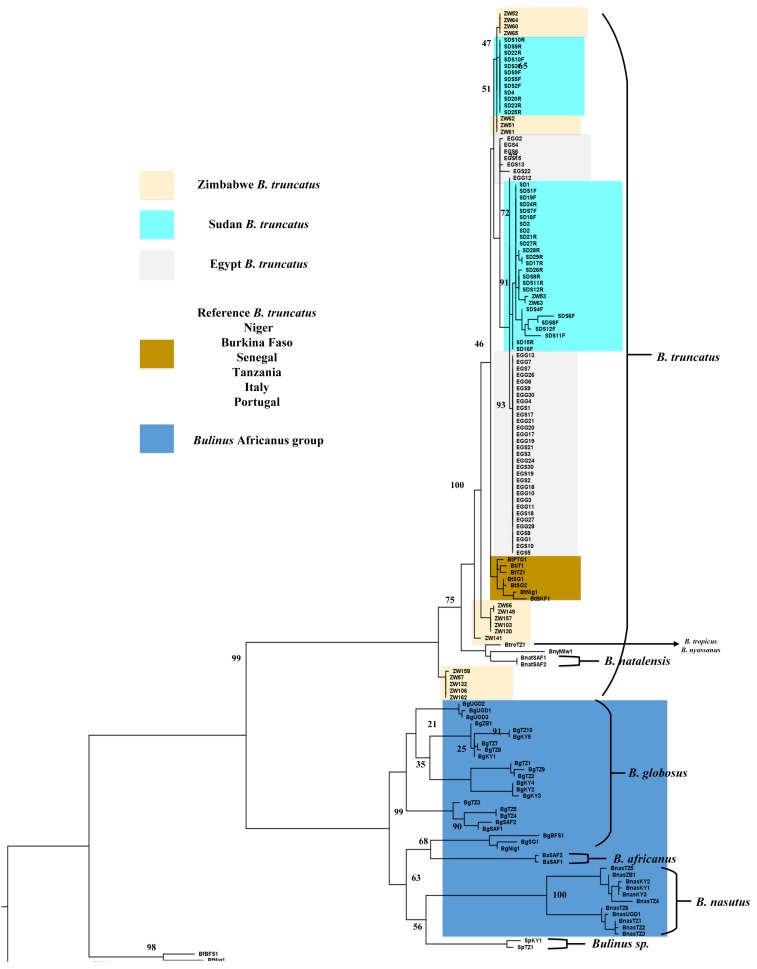
A rooted maximum likelihood tree of *Bulinus truncatus* for CO1 sequences. Maximum likelihood tree of a 737 bp fragment of the cytochrome oxidase subunit 1 (CO1) gene for *B. truncatus* in this study with an additional 51 published Genbank sequences including *B. truncatus* reference isolates. Values on the branches are bootstrap support based on 1000 replications. *B. forskalii* (AM286306.2) was defined as outgroup. * UGD-Uganda, TZ-Tanzania, SAF-South Africa, KY-Kenya, ZB-Zanzibar, SG-Senegal, BFS, Burkina Faso, Nig-Niger, Agl-Angola, Mlw-Malawi.

**Figure 3:**
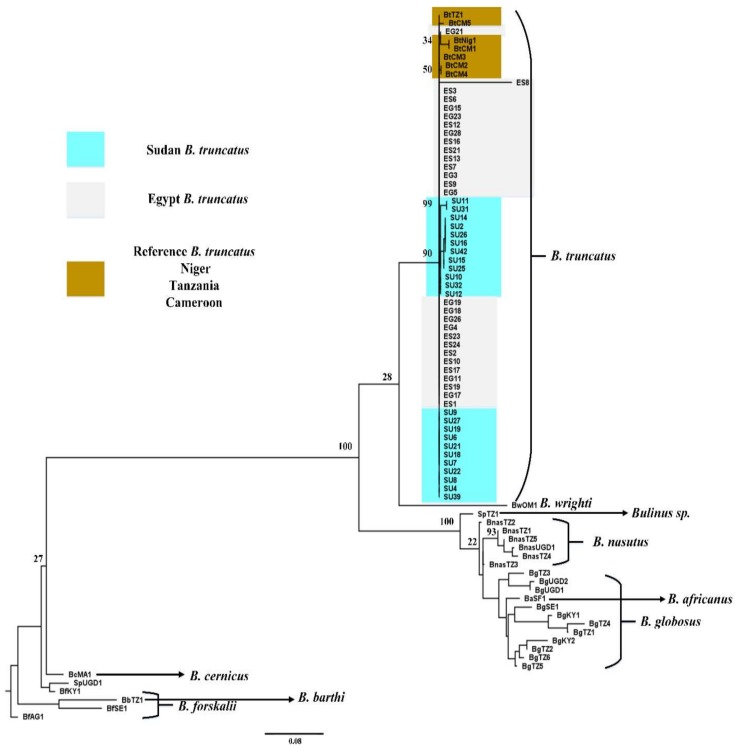
A rooted maximum likelihood tree of *Bulinus truncatus* for ITS 1 sequences. Maximum likelihood tree of a 580 bp fragment of the internal transcribed spacer 1 (ITS 1) for *B. truncatus* in this study with an additional 33 published Genbank sequences including *B. truncatus* reference isolates. Values on the branches are bootstrap support based on 1000 replications. *B. forslkalii* (AM921961.1) was defined as outgroup. * UGD-Uganda, TZ-Tanzania, SF-South Africa, KY-Kenya, SE-Senegal, BFS, Burkina Faso, Nig-Niger, AG-Angola, MA-Mauritius, OM-Oman.

Phylogenetic analyses indicated some measures of variation in the genetic population structure of *B. truncatus* population from Giza and Sharkia (Egypt), Barakat area (Sudan), and Madziwa (Zimbabwe). A large quantity of COI sequence data from Giza and Sharkia (Egypt) and Barakat area (Sudan) cluster together on the *B. truncatus* clade but the COI sequence data of Madziwa (Zimbabwe) *B. truncatus* population cluster at different locations on the tree (Figure 2 and Figure S1). No significant variation was observed between *B. truncatus* populations from Giza and Sharkia (Egypt) and Barakat area (Sudan) (Figure 3 and Figure S2). The minimum spanning tree indicated the torso of *B. truncatus* populations genetic structure (Figure S3). The tree showed five haplotypes for *B. truncatus* obtained from Zimbabwe, while five and two haplotypes were indicated for Sudan and Egypt *B. truncatus* populations respectively. Cryptic lineages or other known species of *B. truncatus* were not detected.

### 3.2. Haplotype and Nucleotide Diversity

Nucleotide polymorphism obtained for all *B. truncatus* sequences assessed in this study is shown in Table 2. Haplotype diversity observed of sequences from the three populations assessed with COI includes 0.334 (Giza and Sharkia, Egypt), 0.768 (Barakat area, Sudan), and 0.784 (Madziwa, Zimbabwe) while their nucleotide diversity includes 0.00205 (s.d. = 0.001445), 0.009359 (s.d. = 0.005067), and 0.014701 (s.d. = 0.007859) for Egypt, Sudan, and Zimbabwe, respectively (Table 2). Haplotype diversity for the two populations assessed with ITS 1 is 0.14 and 0.443 while nucleotide diversity includes 0.005589 (s.d. π 0.003362) and 0.00169 (s.d. π 0.001367) for (Giza and Sharkia, Egypt) and (Barakat area, Sudan), respectively. F_ST_ values in the pairwise population comparisons are shown in Table 3.

## 4. Discussion

Urinary schistosomiasis burden is widely reported in Africa and this is a consequence of the unabated distribution of the important snail intermediate hosts of the genus *Bulinus* that serves as host to the schistosome parasite [4]. The *Bulinus* group is made up of about 37 recognized species and has been divided into four different groups for convenience [5].

This study assessed the genetic diversity of *B. truncatus* populations, the snail host implicated in the transmission of *S. haematobium* in Africa using mitochondrial cytochrome oxidase 1 (COI) gene and internal transcribed spacer (ITS 1).

Snail host identification using morphological characters is unreliable and sometimes ambiguous but the development and application of molecular techniques have been helpful, providing good species discrimination [4,5].

We observed that mitochondrial DNA sequences from Barakat area (Sudan) and Madziwa, Shamva (Zimbabwe) indicated high haplotype diversity including 0.768 and 0.784, respectively, a similar observation was earlier reported by Zein-Eddine et al. [14]. The level of haplotype diversity observed from the two populations in our study is less than the values reported by Zein-Eddine et al. [14]. However, relatively low haplotype diversity was also observed for sequences from Giza and Sharkia (Egypt), with 0.334. Nevertheless, the low levels of haplotype diversity observed for *B. truncatus* in this study were similar to findings by Zein-Eddine et al. [11] and those observed by Goodall-Copestake et al. [35,19], with low diversity species.

Our findings indicated some degree of variation in the *Bulinus* species population structure across Africa. *Bulinus* species populations on both COI and ITS 1 trees (Figure 2 and Figure 3, Figures S1 and S2) separated into populations that correspond to *Bulinus* species groups [12,36]. However, we observed that *B. globosus* from West Africa clusters separately from the East African species as indicated by the COI sequence data.

Kane et al. [12] reported the division between *B. globosus* from the two regions in Africa and that *Bulinus africanus* has a close affinity with West African *B. globosus* species. This is also evident from the information provided by our COI sequence data (Figure 2).

Some levels of segregation were observed within the COI *B. truncatus* populations. The location of populations from Giza and Sharkia (Egypt) and Barakat area (Sudan) on the *B. truncatus* clade agrees with the location of both countries geographically (Figure 2). The clustering of the Madziwa, Shamva (Zimbabwe) sequences in different locations on the *B. truncatus* clade can be attributed to individuals with different genotypes within the population (Figure 2).

Findings from this study using COI identified two reciprocally monophyletic *B. truncatus* sister subclades and this corresponds to *B. truncatus* and *B. tropicus* respectively [12,36]. Nalugwa et al. [36] also obtained similar results from findings on the *B. truncatus/tropicus* complex collected from Albertine Rift freshwater bodies in Uganda. Brown and Shaw [37] have shown that *B. truncatus* is a tetraploid and *B. tropicus* is diploid; however, it is difficult to distinguish *B. truncatus* and *B. tropicus* morphologically.

The minimum spanning network was constructed to support the ML tree and this informed our decision to include some outgroup sequences. The network did not indicate a substantial difference from the information on the tree (Figure S3).

Although *Bulinus* species populations separated distinctly into groups as earlier indicated [12], no significant variation was observed within *B. truncatus* populations from Giza and Sharkia (Egypt) and Barakat area (Sudan) as indicated by the ITS 1 tree (Figure 3). Kane et al. [12] stated that *Bulinus wrighti* has a characteristic COI sequence that positions the species and other members of the *Bulinus reticulatus* group close to the *Bulinus truncatus* complex. This was also observed from our ITS 1 result (Figure 3).

This study is not unique; however, it has investigated the genetic diversity of *B. truncatus* from Giza and Sharkia (Egypt), Barakat area (Sudan), and Madziwa (Zimbabwe), which is necessary for snail host surveillance in the study areas and it has also provided genomic data of this important snail species from the sampled countries.

Although no infection was detected from the snails when screened for patent and prepatent infection, the presence and distribution of *B. truncatus* in the studied areas poses a threat to the inhabitants of these areas should an infected person visit the water bodies and urinate inside or near enough for the schistosome eggs released with the urine to have contact with the water bodies, especially water contact sites such as the Nile River and El-Salam Canal (Egypt), where inhabitants engage in a lot of fishing activities, and the river at Madziwa (Zimbabwe) that people visit frequently to carry out their domestic chores as well as engage in activities such as swimming.

Previous studies implicated *B. truncatus* as the only *Bulinus* species that transmits *S. haematobium* in Egypt and other parts of northern Africa, while *Bulinus globosus* is implicated for schistosomiasis transmission in Zimbabwe [4]. The presence of *B. truncatus* in the southern African country can be attributed to the favorable environmental factors and migration of snail population; however, human activities have also increased the number of snail hosts of *S. haematobium*. This is a cause for concern and there is a need to improve measures for effective snail control strategies.

Differentiating snail host populations to assess their diversity should be prioritized in Africa, where host snails’ genome data is scarce for most schistosomiasis endemic countries [18]. Efforts should be made to initiate a continent-wide snail host genome project to help develop a more comprehensive and robust snail host genome database for the African continent.

## 5. Conclusions

This study identified *B. truncatus,* the snail host of *S. haematobium* obtained from Giza and Sharkia (Egypt), Barakat area (Sudan), and Madziwa, Shamva District (Zimbabwe) using COI and ITS 1 markers, as well as provided information on their genetic diversity.

With the increasing global call that effective schistosomiasis control programmes should target snail control, there is a need to prioritize snail studies for effective mapping of schistosomiasis transmission [38], as well as to strengthen surveillance strategies.

## Figures and Tables

**Table 1:** Geographic coordinates of the study areas.

Country	Location	No. of Samples Collected	Time of Collection	Type of Water Body	Latitude	Longitude
Egypt	Giza (El-Nile river, Gezerite El-Warrak)	25	October, 2016	River	30.102	31.243
	Sharkia (El-Salam canal, El-Hesenia district)	30	November, 2016	Canal	31.258	32.270
Sudan	Barakat area, Wad Madani	14	July, 2016	Canal	14.33673	33.52736
	Barakat area, Wad Madani	22	August, 2016	Canal	14.31780	33.53167
	Barakat area, Wad Madani	5	August, 2016	Canal	14.29210	33.55261
	Barakat area, Wad Madani	8	August, 2016	Canal	14.25122	33.59070
Zimbabwe	Madziwa, Shamva District	11	March, 2016	River	16.93642	31.44603
	Madziwa	6	March, 2016	River	16.91498	31.42868
	Madziwa	10	June, 2016	River	16.85695	31.49413
	Madziwa	3	June, 2016	River	16.88070	31.49083

**Table 2:** Estimation of nucleotide diversity and summary statistics of *Bulinus truncatus* identified using COI I and ITS 1 markers.

		N	H	Hd	π	S.D.π	Θ_S_	s.d.S	Tajima’s D	*p*-Value
COI	Egypt	38	6	0.334	0.00205	0.001445	0.002916	0.001299	−0.85621	0.24
	Sudan	36	11	0.768	0.009359	0.005067	0.009602	0.003341	−0.08761	0.538
	Zimbabwe	20	6	0.784	0.014701	0.007859	0.011655	0.004442	1.01745	0.898
ITS 1	Egypt	28	3	0.14	0.005589	0.003362	0.020107	0.006909	−2.69592	0
	Sudan	23	2	0.443	0.00169	0.001367	0.001034	0.000769	1.41416	0.923

Number of sequences (N), number of haplotypes (h), haplotype diversity (Hd), nucleotide diversity (pi), theta per site (Θ_S_), standard deviation (s.d.).

**Table 3:** Population pairwise Φst.

	Egypt	Sudan
Sudan	0.34860 *	
Zimbabwe	0.59653 *	0.36160 *

* Significant *p* value < 0.05.

## Supplementary Materials

The following are available online at http://www.mdpi.com/2414-6366/3/4/127/s1. S1A–S1J, data were used to prepare Table 2, S1A: Egypt B. truncatus COI.out, S1B: Egypt B. truncatus COI Tajima D.out, S1C: Egypt B. truncatus Fu_Li Test.out, S1D: Egypt_SUD_ZWE B. truncatus COI final.out, S1E: Fu_Li Egypt_SUD_ZWE B. truncatus COI.out, S1F: Sudan B. truncatus COI.out, S1G: Sudan B. truncatus COI Tajima D.out, S1H: Sudan B. truncatus Fu_Li test.out, S1I: Zimbabwe B. truncatus.out, S1J: Zimbabwe B. truncatus COI Tajima D.out, S2A–S2F data were used to prepare Table 3, S2A: Egypt B. truncatus ITS Fu_Li test.out, S2B: Egypt B. truncatus ITS Tajima D.out, S2C: Egypt B. truncatus ITS.out, S2D: Sudan B. truncatus ITS.out, S2E: Sudan B. truncatus ITS Fu_Li test.out, S2F: Sudan B. truncatus ITS Tajima D.out, S3A–S3M were used to prepare Figure 2A,B, S3A: Egypt Bulinus truncatus GENBANK PROTEIN SEQ NEW.fas, S3B: Egypt Bulinus truncatus GENBANK SEQ NEW.d.txt, S3C: Egypt B. truncatus GENBANK SEQ NEW.fas, S3D: SUDAN B. truncatus COI Protein SEQ1.fas, S3E: SUDAN B. truncatus COI genbankSEQ.fas, S3F: SUDAN B. truncatus Genbank SEQ COI.txt, S3G: Zimbabwe B.truncatus COI protein SEQ for genbank submission.fas, S3H: Zimbabwe B. truncatus COI SEQ for genbank submission.fas, S3I: Zimbabwe B. truncatus COI SEQ for genbank submission.txt, S3J: ZWE_SUDAN_EGYPT_BT_REF_COI_OUTGROUP.txt, S3K.ZWE_SUDAN_EGYPT_BT_REF_COI_OUTGROUP2-v2NEW.fas, S3L: Summary of the STRUCTURE analysis for B. truncatus COI.docx, S3M: Table 1. Information on B. truncatus sequences submitted to genbank, S4A–S4H were used to prepare Figure 3A,B, S4A: Information B. truncatus ITS 1 sequences submitted to genbank.xls, S4B: Egypt B. truncatus ITS.fas, S4C: Egypt B. truncatus ITS.txt, S4D: Egypt_Sudan_REF B. truncatus ITS.fas, S4E: Egypt_Sudan_REF_Outgroup B. truncatus ITS.txt, S4F: SUDAN B. truncatus ITS.fas, S4G: SUDAN B. truncatus ITS.txt, S4H: Summary of STRUCTURE analysis for B truncatus.docx.
