# Supplementary material for: Phylogeography of Bulinus truncatus (Audouin, 1827) (Gastropoda: Planorbidae) in Selected African Countries
Source: Trop Med Infect Dis. 2018 Dec 19;3(4):127. doi: 10.3390/tropicalmed3040127 (PMC6306716; doi:10.3390/tropicalmed3040127)
Supplement: Supplementary file 1 [file tropicalmed-03-00127-s001.zip › Supplementary data3/ML Bayes trees illustration.docx]

**Figure S1.**  A rooted maximum likelihood Bayes phylogenetic tree of *Bulinus truncatus* for CO1 sequence. Maximum likelihood tree of a 737 bp fragment of the cytochrome oxidase subunit 1 (CO1) gene for *B. truncatus* in this study and additional 51 published Genbank sequences including *B. truncatus* reference isolates. Values on the branches are bootstrap support based on 1000 replications. *B. forskalii* (AM286306.2, AM286308 and AM 286310) were defined as outgroup.

**Figure S2.** A rooted maximum likelihood Bayes phylogenetic tree *Bulinus truncatus* for ITS 1 sequence. Maximum likelihood tree of a 580 bp fragment of the Internal Transcribed Spacer 1 (ITS 1) for *B. truncatus* in this study with additional 33 published Genbank sequences including *B. truncatus* reference isolates. Values on the branches are bootstrap support based on 1000 replications. *B. forslkalii* (AM921961.1) was defined as outgroup.
