# Supplementary figures and images for: Phylogeography of Bulinus truncatus (Audouin, 1827) (Gastropoda: Planorbidae) in Selected African Countries
Source: Trop Med Infect Dis. 2018 Dec 19;3(4):127. doi: 10.3390/tropicalmed3040127 (PMC6306716; doi:10.3390/tropicalmed3040127)

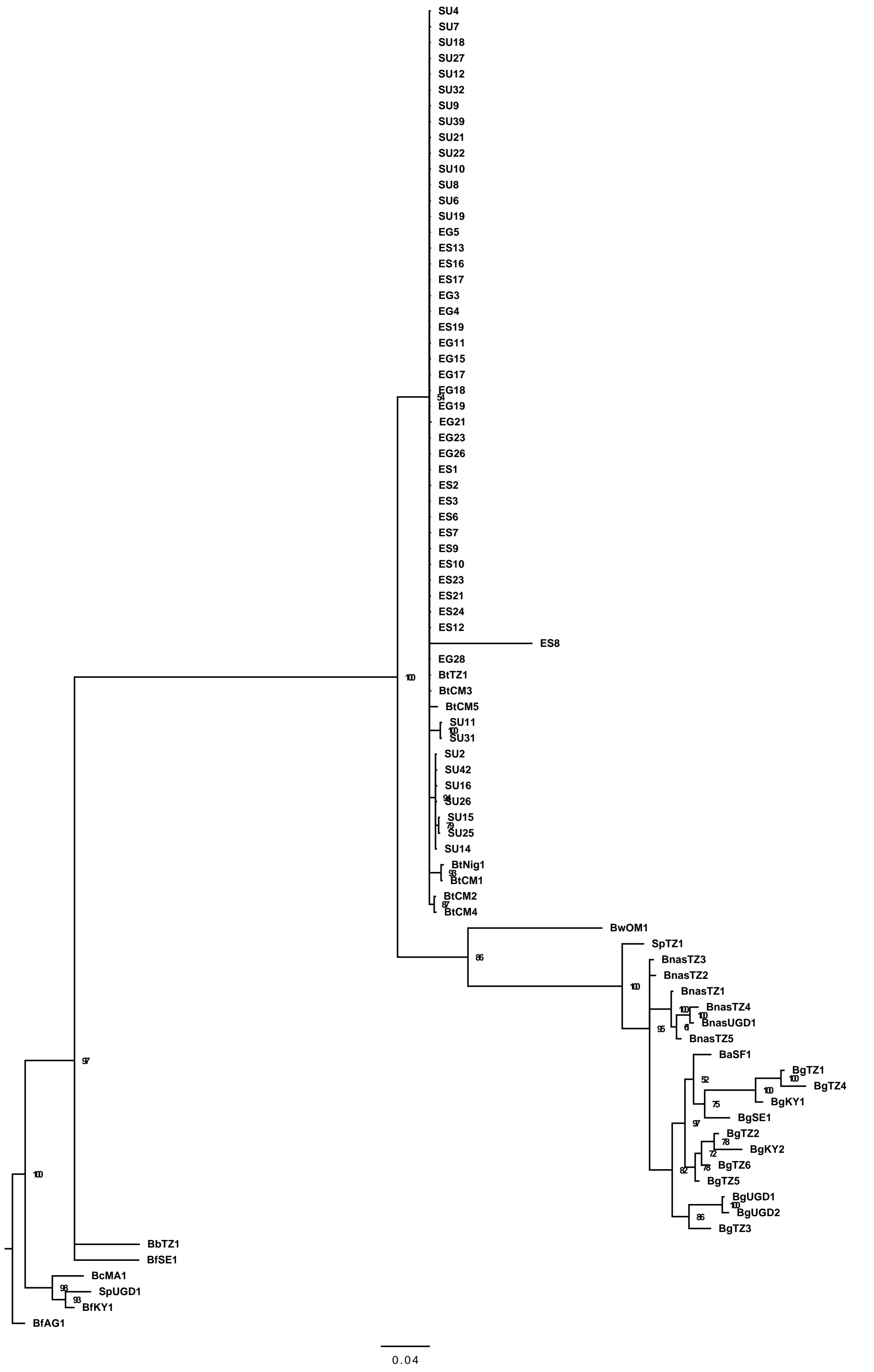

Supplement: Supplementary file 1 [file tropicalmed-03-00127-s001.zip › Supplementary data3/Figure S2_.ML Bayes Phylogenetic tree for ITS 1 Bulinus truncatus .pdf]

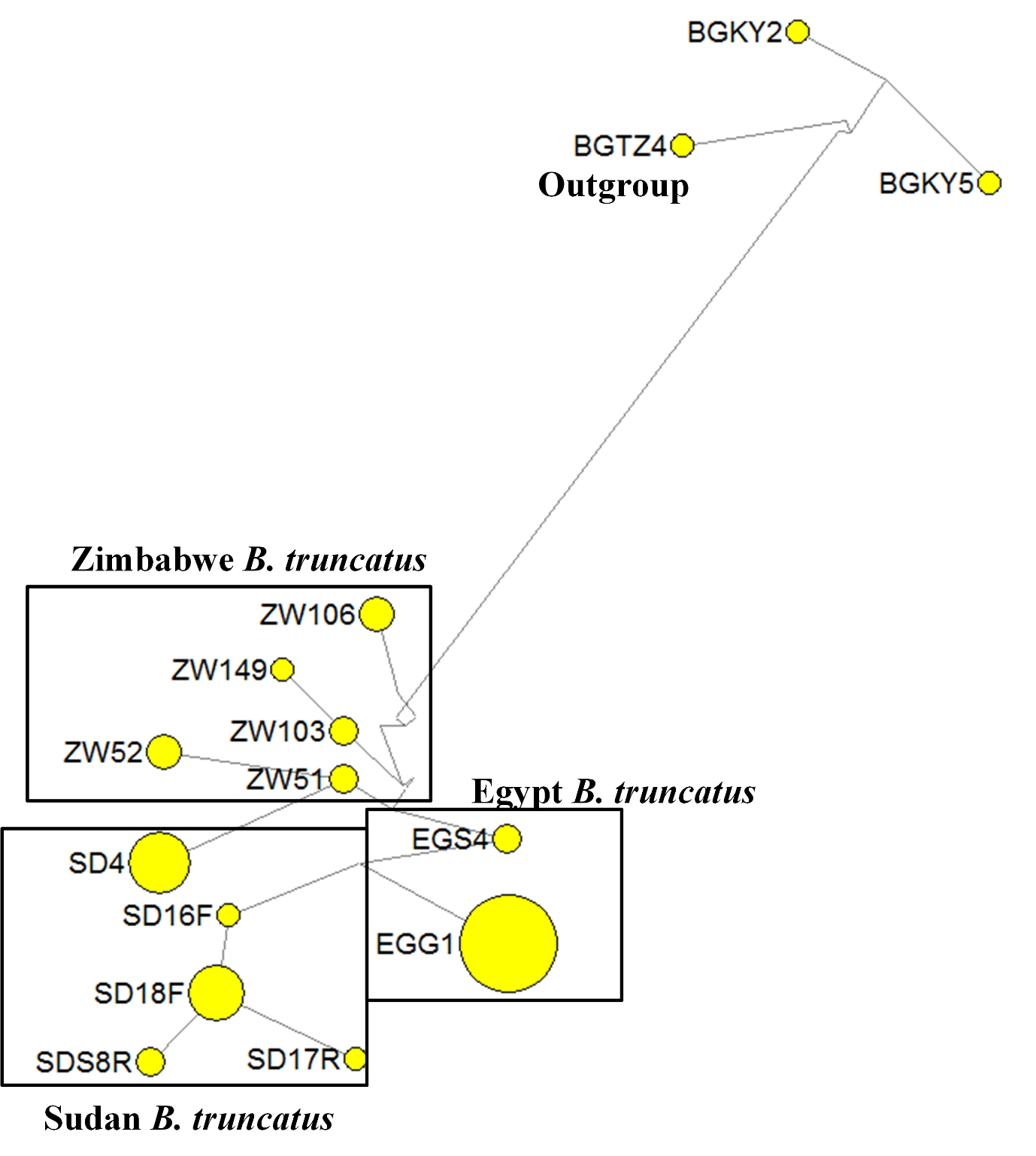


Figure S3: Minimum spanning network for CO1 *B. truncatus* populations

Supplement: Supplementary file 1 [file tropicalmed-03-00127-s001.zip › Supplementary data3/Figure S3_ Neighbour joining network for CO1 B. truncatus populations.docx]
