# Supplementary material for: Phylogeography of Bulinus truncatus (Audouin, 1827) (Gastropoda: Planorbidae) in Selected African Countries
Source: Trop Med Infect Dis. 2018 Dec 19;3(4):127. doi: 10.3390/tropicalmed3040127 (PMC6306716; doi:10.3390/tropicalmed3040127)
Supplement: Supplementary file 1 [file Figure S1_ ML Bayes Phylogenetic tree for COI B truncatus.pdf › Supplementary data3/Figure S1_ ML Bayes Phylogenetic tree for COI B truncatus.pdf]

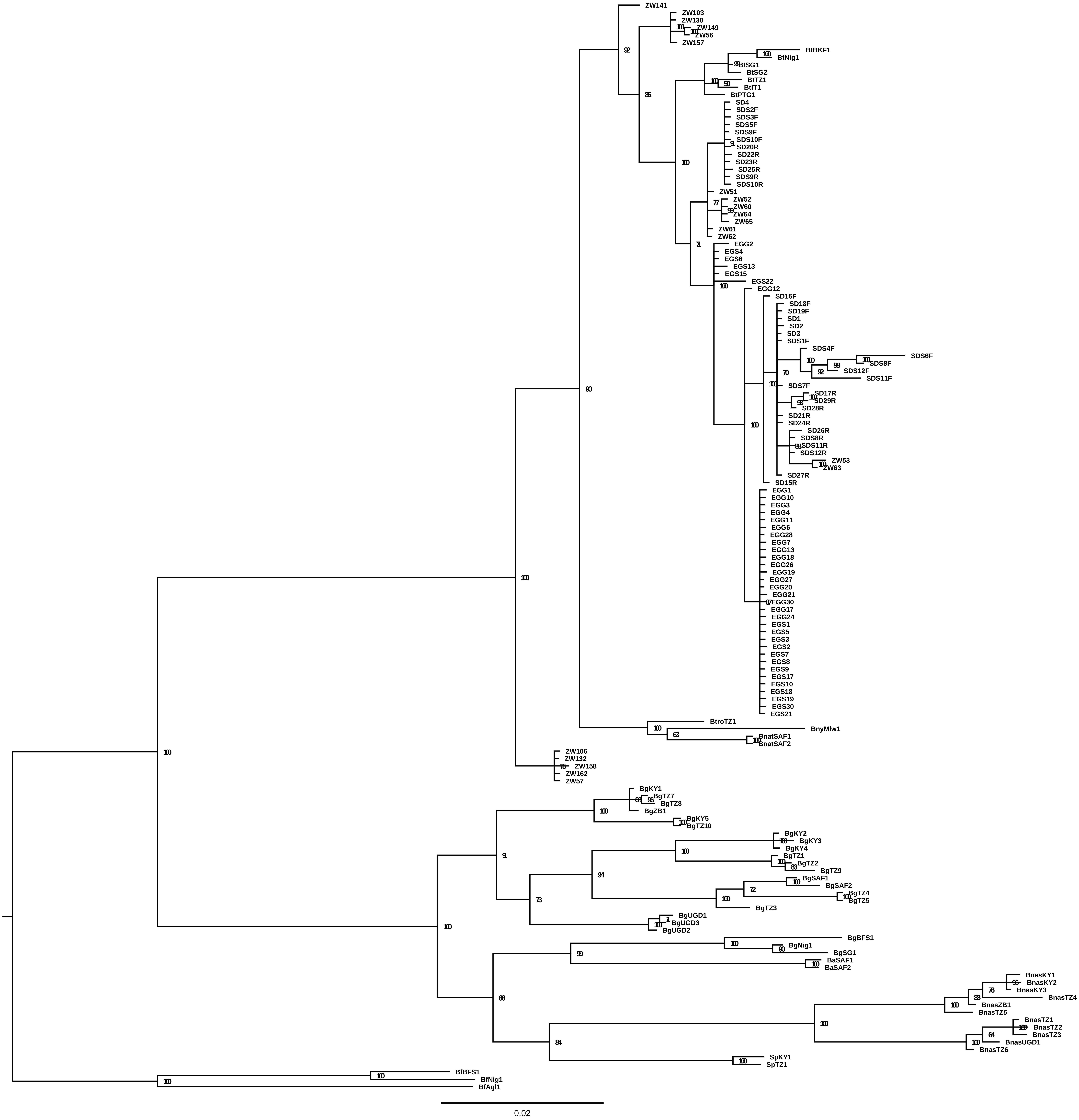
ZW141
ZW103
ZW130
ZW149
ZW56
ZW157
BtBKF1
BtNig1
BtSG1
BtSG2
BtTZ1
BtIT1
BtPTG1
SD4
SDS2F
SDS3F
SDS5F
SDS9F
SDS10F
SD20R
SD22R
SD23R
SD25R
SDS9R
SDS10R
ZW51
ZW52
ZW60
ZW64
ZW65
ZW61
ZW62
EGG2
EGS4
EGS6
EGS13
EGS15
EGS22
EGG12
SD16F
SD18F
SD19F
SD1
SD2
SD3
SDS1F
SDS4F
SDS6F
SDS8F
SDS12F
SDS11F
SDS7F
SD17R
SD29R
SD28R
SD21R
SD24R
SD26R
SDS8R
SDS11R
SDS12R
ZW53
ZW63
SD27R
SD15R
EGG1
EGG10
EGG3
EGG4
EGG11
EGG6
EGG28
EGG7
EGG13
EGG18
EGG26
EGG19
EGG27
EGG20
EGG21
EGG30
EGG17
EGG24
EGS1
EGS5
EGS3
EGS2
EGS7
EGS8
EGS9
EGS17
EGS10
EGS18
EGS19
EGS30
EGS21
BtroTZ1
BnyMlw1
BnatSAF1
BnatSAF2
ZW106
ZW132
ZW158
ZW162
ZW57
BgKY1
BgTZ7
BgTZ8
BgZB1
BgKY5
BgTZ10
BgKY2
BgKY3
BgKY4
BgTZ1
BgTZ2
BgTZ9
BgSAF1
BgSAF2
BgTZ4
BgTZ5
BgTZ3
BgUGD1
BgUGD3
BgUGD2
BgBFS1
BgNig1
BgSG1
BaSAF1
BaSAF2
BnasKY1
BnasKY2
BnasKY3
BnasTZ4
BnasZB1
BnasTZ5
BnasTZ1
BnasTZ2
BnasTZ3
BnasUGD1
BnasTZ6
SpKY1
SpTZ1
BfBFS1
BfNig1
BfAgl1
0.02
